# 8-Bit Adder and Subtractor with Domain Label Based on DNA Strand Displacement

**DOI:** 10.3390/molecules23112989

**Published:** 2018-11-15

**Authors:** Weixuan Han, Changjun Zhou

**Affiliations:** 1 College of Mathematics and Computer Science, Zhejiang Normal University, Jinhua 321004, China; m18340852618@163.com; 2 College of Nuclear Science and Engineering, Sanmen Institute of technicians, Sanmen 317100, China; 3 Key Laboratory of Advanced Design and Intelligent Computing (Dalian University) Ministry of Education, Dalian 116622, China

**Keywords:** DNA strand displacement, cascade, 8-bit adder/subtractor, domain label

## Abstract

DNA strand displacement, which plays a fundamental role in DNA computing, has been widely applied to many biological computing problems, including biological logic circuits. However, there are many biological cascade logic circuits with domain labels based on DNA strand displacement that have not yet been designed. Thus, in this paper, cascade 8-bit adder/subtractor with a domain label is designed based on DNA strand displacement; domain t and domain f represent signal 1 and signal 0, respectively, instead of domain t and domain f are applied to representing signal 1 and signal 0 respectively instead of high concentration and low concentration high concentration and low concentration. Basic logic gates, an amplification gate, a fan-out gate and a reporter gate are correspondingly reconstructed as domain label gates. The simulation results of Visual DSD show the feasibility and accuracy of the logic calculation model of the adder/subtractor designed in this paper. It is a useful exploration that may expand the application of the molecular logic circuit.

## 1. Introduction

In recent years, biological computing has become a new hotspot due to DNA molecules having the advantages of parallelism, low energy consumption, and high storability in dealing with massive information; therefore, DNA nanotechnology stands out DNA nanotechnology has potential applications in biological calculations. A range of information circuits and bio-computing models have been implemented in DNA by using strand displacement. Examples include DNA strand displacement reactions [1,2], molecular motors [3,4,5], catalytic signal amplification circuits [6,7,8], and biological logic circuits [9,10,11], as well as computing with membranes, bacteria, conjugation and RNA computing [12,13,14,15,16]. As a new technique in the field of self-assembled DNA, the DNA strand displacement reaction has been widely used in the field of molecular computing. DNA molecular circuits corresponding to different logic gates have been designed on the basis of the DNA self-assembly calculation principle. When the DNA signal strand is input into the molecular logic circuit, molecular logic gates with different molecular concentration ratios are mixed, and the molecular logic circuit outputs the signal strand through intermolecular specific hybridization and the DNA strand displacement reaction. In 2006, Seeling designed the AND Gate, OR Gage, NOT Gate signal amplifier and signal feedback using single-stranded nucleic acids as the input and output signals based on DNA strand displacement [17]. However, NOT Gate output is unstable due to its single-stranded input. In 2011, Qian designed a simple Seesaw logic gate, four-bit square root biological logic circuit and avoided the problem of NOT Gate output instability via the use of dual-rail [18,19]. However, dual-rail logic is not suitable for large-scale cascaded molecular logic circuits. In 2013, Zhang proposed and verified the logical “AND” gate and “OR” gate [20]. In 2014, Guo designed multiple types of logic gate based on a single g-quadrupled DNA strand [21]. In 2015, Wang used strand displacement to achieve the multi-bit adder design [22]. In 2016, Lakin presented a framework for the development of adaptive molecular circuits using buffered DNA strand displacement networks and designed supervised learning in adaptive DNA strand displacement networks [23]. In 2017, Sun presented a one-bit half adder-half subtractor logical operation based on DNA strand displacement [24].

Although Winfree [19] solved the instability caused by NOT Gate and then designed many stable biological logic circuits, dual-rail logic brought many new problems. The scale of a dual-rail logic circuit is two times that of a single-rail logic circuit, which increases the material cost, complexity, and difficulty of designing the logic circuit. The most fundamental problem of the instability caused by NOT Gate and the scale of the dual-rail logic circuit is that concentration is applied to the presentation of logic 1 and logic 0. The concentration of a reactant has an important effect on the reaction rate, because the reaction rate of high concentration reactants is faster than that of low concentration reactants, and a reaction works from an area of high concentration to an area of low concentration under the same circumstances [25]. It appears that changing the conditions of the NOT Gate of a single-rail logic circuit goes against the design of a large-scale single-rail logic circuit.

In this paper, DNA signals are marked with domain labels based on the freedom of DNA hybridization and the high sensitivity of domain labels, and then logic gates with domain labels are constructed by redesigning the special DNA structure, where logic value 1 and logic value 0 are represented by domain t and domain f, respectively, which solves the instability issue of NOT Gate in dual-rail logic. Based on this, the first domain label, cascade 8-bit adder/subtractor is designed. The innovation of this paper is that the design of the molecular domain label is used in the logic gates, where logical results are detected through the labeling of the domain label with a fluorescent label in the reaction solution. This was not seen in the previous design of the molecular logic circuit, and this paper broadens the range of input signals with DNA molecules to construct the logic circuit. The detection method of the logic gate model has high sensitivity and simple operation. It has less stringent requirements for base mismatches, reducing the impact of hybrid competition in the experimental results to a certain extent. In addition, the domain label cascade 8-bit adder/subtractor can be used to design large scale biochemical circuits to allow good encapsulation.

This paper is arranged as follows: the development of DNA molecule logic circuits is introduced in the first part; the background of DNA strand displacement and logic gates is presented in the second part; a brief method for building domain label logic gates is presented in the third part; the simulation of the domain label cascade 8-bit adder/subtractor by Visual DSD is presented in the fourth part; and the fifth part presents the conclusions of this paper.

## 2. Backgrounds

### 2.1. DNA Strand Displacement Reaction

**Figure 1:**
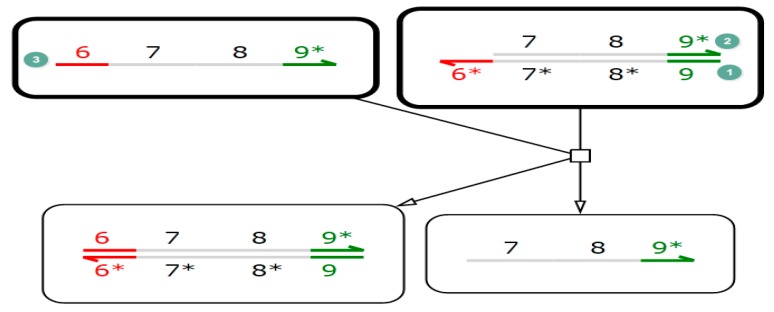
DNA strand displacement.

Utilizes the characteristics of the free energy of the molecular hybridization system to stabilize the state and control or induce downstream strand displacement reactions by changing the sequence and length of the input signal. Intuitively, DNA strand displacement is the process of replacing a shorter hybridization region with a longer, double-stranded hybridization region. The process of this is shown in Figure 1 [26].

The process of the reversible DNA strand displacement reaction is shown in Figure 1. Firstly, two partially complementary DNA strands are joined together (the 1-strand is longer than the 2-strand), Secondly, the 3-strand is added to the solution at room temperature (the sequence of the 3-strand is completely complementary to the 1-strand). Thirdly, the specific recognition region is first combined with a single strand of the 1-strand. In order to achieve the most stable state, the binding sites of the 1-strand and 2-strand are gradually occupied by the 3-strand, and finally, the 3-strand completely replaces the 2-strand. As the DNA strand displacement reaction with highly specific identification sites can start in parallel and realize a multi-level nested trigger, it has developed rapidly in recent years and has become a hotspot in the field of molecular computing.

### 2.2. DNA Logic Module with Domain Label

**Figure 2:**
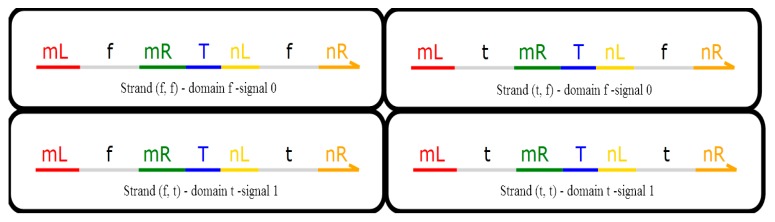
Domain f and domain t.

On the basis of the existing DNA logic model, the domain label is used to realize the operation of the domain label logic module which can react spontaneously at room temperature. Domain t and domain f respectively represent signal 1 and the signal 0, which correspond to the regions of high concentration and low concentration in the solution, which are shown in Figure 2 [27].

As can be seen from Figure 2, a domain label DNA signal strand consists of a left domain and a right domain, where the right domain is responsible for passing the logic signal to the downstream logic gate, and the left domain is responsible for receiving the upstream DNA signal. Therefore, the (f, t) and (t, t) strands are known as domain t and correspond to signal 1 while the (f, f) and (t, f) strands are known as domain f and correspond to signal 0.

**Figure 3:**
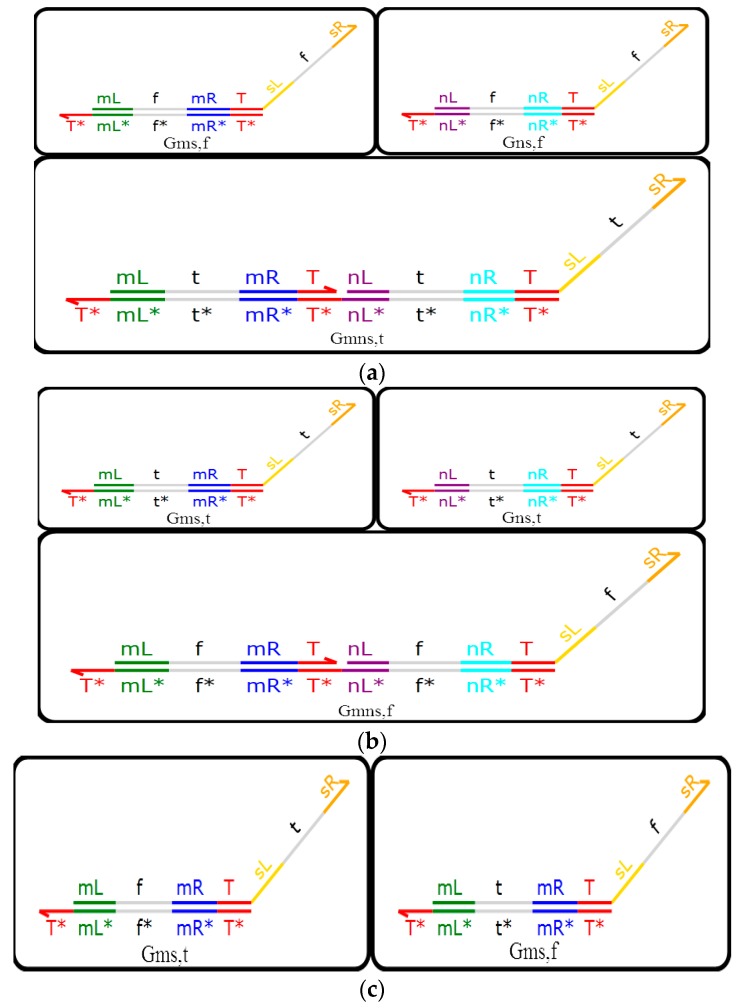
(**a**) The AND Gate module with a domain label; (**b**) the OR Gate module with a domain label; (**c**) the NOT Gate module with a domain label.

The AND Gate with a domain label, the OR Gate with a domain label and the NOT Gate with a domain label are the most elementary logic modules, the logic gates of which are shown in Figure 3. The AND Gate with a domain label and the OR Gate with a domain label are made up of three DNA double strands. The NOT Gate with a domain label consists of two DNA double strands. In Figure 3a, AND Gates with domain labels are denoted by $G_{\mathrm{ms},f},\;$ $G_{\mathrm{ns},f}$ and $G_{\mathrm{mns},t}$ respectively. In Figure 3b, OR Gates with domain labels are denoted by $G_{\mathrm{ms},t}$, $G_{\mathrm{ns},t}$ and $G_{\mathrm{mns},f}$ respectively In Figure 3c, the two DNA double strands are the same, except for the locations of t and f of the NOT Gate with a domain label.

Whether a DNA single strand represents logic 1 or logic 0 depends on its presence (domain t~logic value 1, domain f~logic value 0). When two strands with domain t are input to the AND Gate with a domain label at the same time, the DNA molecule reaction depends on $G_{\mathrm{mns},t}$, and finally, it outputs a strand with domain t; in other cases, the DNA molecule reaction depends on o, $G_{\mathrm{ms},f}$ or $G_{\mathrm{ns},f},$ and finally, it outputs a strand with domain f. When two strands with domain f are input to the OR Gate with a domain label at the same time, the DNA molecule reaction depends on $G_{\mathrm{mns},f}$, and finally, it outputs a strand with domain f; in other cases, the DNA molecule reaction depends on $G_{\mathrm{ms},t}$ or $G_{\mathrm{ns},t}$, and finally, it outputs a strand with domain t. As for the NOR Gate, the situation is much simpler. When a strand with domain f is input to the NOR Gate with a domain label, the DNA molecule reaction depends on $G_{\mathrm{ms},t}$, and finally, it outputs a strand with domain t; otherwise, the DNA molecule reaction depends on $G_{\mathrm{ms},f}$, and finally, it outputs a strand with domain f.

### 2.3. Mapping

K operation: Let A be a non-empty set. The Cartesian product $A^{K}=A\times A\times A\times \cdots \times A$ to A mapping f is called the N operation on the set A. In addition, each element in $\left|A^{K}\right|$ has |A| possible correspondences of |A|, so from $A^{K}$ to A, it has ${\left|A\right|}^{{\left|A\right|}^{K}}$ possible mappings, which is called m, m = $m={\left|A\right|}^{{\left|A\right|}^{K}}$. K = 1 is called 1-input mapping. K = 2 is called 2-input mapping. If A=[0,1], n = 1, then $m={\left|A\right|}^{{\left|A\right|}^{K}}=2^{2^{1}}=4$. n = 2, then $m={\left|A\right|}^{{\left|A\right|}^{K}}=2^{2^{2}}=16$, and so on. If each N-input mapping module is a mapping from $A^{K}={\left\{t,f\right\}}^{K}$ to $A^{K}={\left\{t,f\right\}}^{K}$, there are a total of ${\left|A\right|}^{{\left|A\right|}^{K}}=2^{2^{K}}$ mappings, so K-input mapping has $2^{2^{K}}$ modules.

## 3. Methods

In this paper, the logic circuit domain label and double-dual are briefly compared in terms of the stability of the molecular reaction process. In the double-dual logic circuit, the Seesaw module contains threshold gates which react with the upstream DNA signal strands irreversibly. The next step of the molecular reaction can be carried out only the threshold gate is completely consumed, so theoretically, the smaller the concentration of the threshold is, the faster the speed of reaction is. The significance of the threshold is to distinguish between the high and low concentrations of the reaction process so that the logical values 1 and 0 are correctly expressed. The double-dual logic circuit corresponds to logic 1 and logic 0 via the high concentration and low concentration, respectively. In general, a DNA signal strand with a unit concentration of 0.9~1 (1 unit concentration of 10,000 nM in this paper) represents logic value 1, and a DNA signal strand with a unit density of 0~0.1 represents logic value 0. It is worth noting that the threshold can correctly express the logical value at the average concentration value, but the molecular reaction is extremely unstable at the threshold concentrations of 0.1~0.2 units and 0.8~0.9 units. In such cases, the threshold cannot strictly distinguish between high concentration and low concentration in the reaction process, resulting in the output of the wrong signal, while logic circuits with domain t and domain f are determined throughout the reaction process and will not output the wrong signal, thereby avoiding the instability of the molecular circuit in the reaction process. On this basis, the paper constructed an N-mapping module with a domain label, an amplification gate with a domain label, a fan-out gate with a domain label, and a reporter gate with a domain label. This formed the basis for constructing the 8-bit addition and subtraction with a domain label.

### 3.1. N-Mapping Module with Domain Label

**Figure 4:**
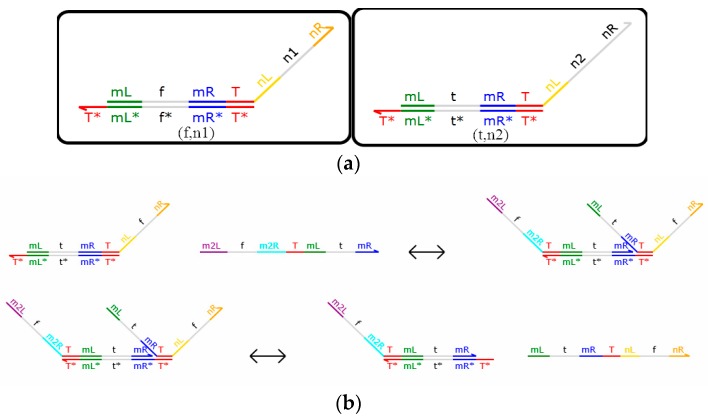
(**a**) 1-Mapping module.; (**b**) Reaction of the 1-mapping module.

The 1-input mapping module consists of strands with the domain label {T^*} [mL^ f mR^ T^] <nL^ n1 nR^> and {T^*} [mL^ t mR^ T^] <nL^ n2 nR>, which note (f, n1), (t, n2). As shown in Figure 4a, there are 4 corresponding mapping modules (n1 = t, n2 = f; n1 = f, n2 = t; n1 = f, n2 = f; n1 = t, n2 = t). It is noteworthy that, when n1 = t, n2 = f, the DNA strands output the opposite domain label signal, and the mapping module implements the NOT gate function. The reaction is shown in Figure 4b. Generally, this function is commonly used in domain label 1 input module mapping.

As shown in Figure 4a, although the 1-mapping module consists of two DNA strands with the same structure and initial concentration, the DNA signal strand actually reacts with one of them, and the reaction is accomplished within only one step. The 1-mapping module shown in Figure 4b implements the NOT operation, in which case n1 = t, n2 = f, and it transforms logic value 1, which is represented by <m2L^ f m2R^ T^mL^ t mR^>, to logic value 0, which is represented by <mL^ t mR^ T^ nL^ f nR^>. The remaining double strands, {T ^*} [mL ^ f mR ^ T ^] <nL ^ t nR>, follow the same principle. A single strand containing domain labels <m2L^ t m2R^ T^ mL^ f mR^> is input, and this reacts with {T ^*} [mL ^ f mR ^ T ^] <nL ^ n1 nR> and outputs logic value 1, which is represented by <mL^ f mR^ T^ nL^ t nR^>.

Therefore, when double strands with domain labels n1 and n2 are inverted (n1 = t, n2 = f; n1 = f, n2 = t), the NOT operations are performed with each of them at the same time, which makes the reaction proceed simultaneously and reduces the time consumed by the reaction. The advantages of the modules are demonstrated here.

The K-input mapping module consists of $2^{K}$ strands with domain labels, namely, {T^*} [m1L^ f m1R^ T^]:…:[mkL^ f mkR^ T^] <nL^ n1 nR^>, {T^*} [m1L^ f m1R^ T^]:…:[mkL^ t mkR^ T^] <nL^ n2 nR^>,…, and {T^*} [m1L^ t m1R^ T^]:…:[mkL^ t mkR^ T^] <nL^ $n2^{K}$ nR^>, Among them (ff,…f, n1), (f…f, t, n2),…, and (t…t, t, $n2^{K}$)., n1, n2… $n2^{K}\in \{t,f\}$. There are $2^{2^{K}}$ corresponding mapping modules, and similar to the 1-input mapping module, the K-input mapping module can also perform different logic operations under k inputs and produces one output. In this paper, we describe 1, 2, and 3-input mapping modules.

The 2-input mapping module consists of strands with domain labels {T^*} [mL^ f mR^ T^]: [nL^ f nR^ T^] <hL^ n1 hR^>, {T^*} [mL^ f mR^ T^]: [nL^ t nR^ T^] <hL^ n2 hR^>, {T^*} [mL^ t mR^ T^]: [nL^ f nR^ T^] <hL^ n3 hR^>, and {T^*} [mL^ t mR^ T^]: [nL^ t nR^ T^] <hL^ n4 hR^> Among them (ff, n1), (ft, n2), (tt, n3), and (tf, n4). As shown in Table 1, there are 16 corresponding mapping modules, namely, (ff, n1)~(ff, n1); (ff, n1)~(ft, n2); (ff, n1)~(tf, n3); (ff, n1)~(tt, n4); (ft, n2)~(ft, n2); (ft, n2)~(ff, n1); (ft, n2)~(tft, n3); (ft, n2)~(tt, n4); (tf, n3)~(tf, n3); (tf, n3)~(ff, n1); (tf, n3)~(ft, n2); (tf, n3)~(tt, n4); (tt, n4)~(tt, n4); (tt, n4)~(ff, n1); (tt, n4)~(ft, n2); (tt, n4)~(tf, n3). It is noteworthy that, when n1 = n2 = n3 = f, n4 = t, only two single strands need to be input, then the AND gate logic can be realized; when n1 = f, n2 = n3 = n4 = f = t, the input of two single strands can implement OR gate logic; when n1 = n4 = f, n2 = n4 = t, only two single strands need to be inputted for XOR gate logic to be realized, which is called a 2-input 1-output mapping module. Generally, this function is commonly used for 2-input module mapping.

Therefore, when n1 = n4 = f, n2 = n4 = t, XOR logic can be realized, which is used as an n-bit adder with a domain label. Generally, the reactions proceed simultaneously, reducing the time consumed by the reaction. The advantages of the modules are demonstrated again.

### 3.2. Amplification Gate with Domain Label

**Figure 5:**
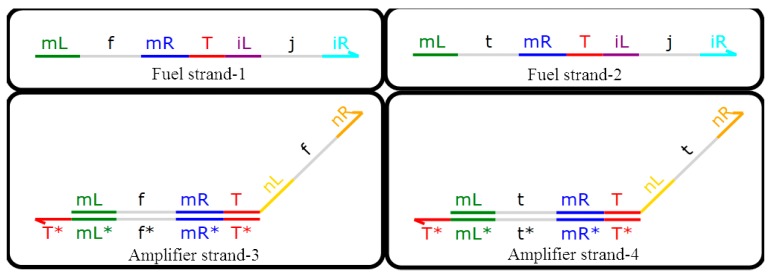
Amplification gate with a domain label.

DNA signals will be attenuated during the reaction process, and lower concentrations will affect the rate of the DNA reaction and the final detection accuracy, so an amplification gate with a domain label was designed in this paper, consisting of two DNA double strands with domain labels and two DNA single strands with domain labels, namely, {T^*} [mL^ f mR^ T^] <nL^ f nR^>, {T^*} [mL^ t mR^ T^] <nL^ t nR^>, and <mL^ f mR^ T^ iL^ jiR^>, <mL^ t mR^ T^ iL^ jiR^>, which corresponds to amplifier strand-3, amplifier strand-4, fuel strand-1, and fuel strand-2. As shown in Figure 5, in the amplifier, the two single strands are of the same concentration and act as fuel during the DNA reaction. Of course, the two double strands also have the same concentration, and the total concentration of the DNA single strand with a domain label is always set to two times that of double strands with a domain label.

**Figure 6:**
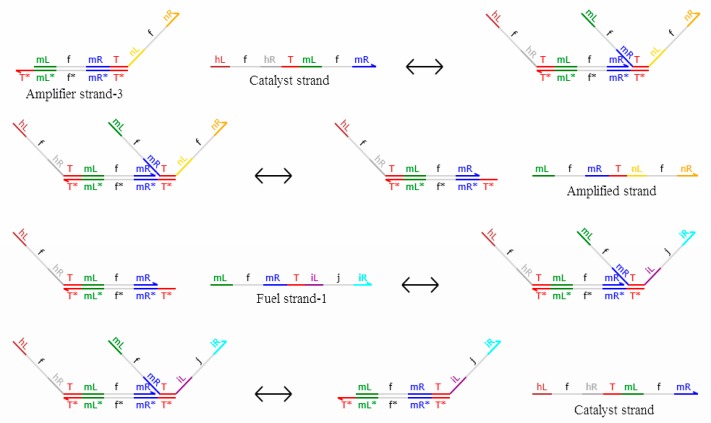
Reaction between fuel strand-1and amplifier strand-3.

It is noteworthy that the domain label is a long domain so only two DNA strands with domain labels are involved in the reaction, amplifying the concentration of the upstream DNA signal strand with a domain label to a set value (one-unit concentration in this paper). Namely, fuel strand-2 and amplifier strand-4 will react with the upstream DNA signal strand that represents logic value 1, and the other two strands do not participate in the reaction. Similarly, fuel strand-1 and amplifier strand-3 will react with the upstream DNA signal strand that represents logic value 0, and the other two strands do not participate in the reaction. In summary, one and only one of the above situations occurs when the DNA system is being reacted. More specifically, for DNA signal strands with a domain label that represents a logical value of 0, the DNA single strands <mL^ t mR^ T^ nL^ t nR^> cannot appear in the output strands. For DNA signal strands with a domain label that represents a logical value of 1, it is also unlikely that the DNA single strands <mL^ f mR^ T^ nL^ fnR^> will appear in the output strands. The reaction between the amplification gate with a domain label and the DNA signal strand with a domain label is shown in Figure 6. Compared with the amplifier of DNA dual-track logic circuit, the results of domain-labeled DNA reaction systems have better certainty.

Figure 6 shows fuel strand-1 and amplifier strand-3 as the reaction strands. <mL^ f mR^ T^ nL^ f nR^> as the output strands, which are called amplified strands and have a logic value of 0; <hL^ f hR^ T^ mL^ f mR^> as the catalytic strand; and other strands as the middle process reaction strands. When fuel strand-1 and amplifier strand-3 participate in the reaction, fuel strand-2 and amplifier strand-4 are invalid. Similarly, when fuel strand-2 and amplifier strand-4 participate in the reaction, fuel strand-1 and amplifier strand-3 are invalid, and the logic value of the output strand is 1.

### 3.3. Fan-Out Gate with Domain Label

**Figure 7:**
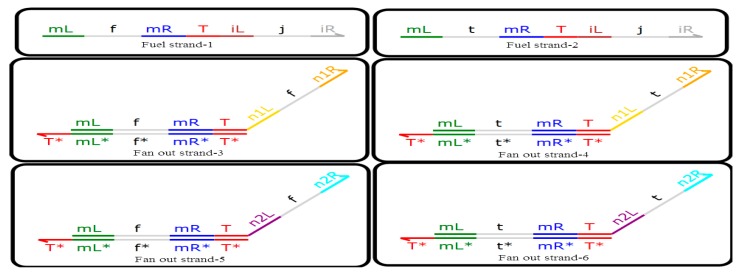
2-Fan out gate with a domain label.

The functions of the fan-out gate with a domain label and the fan-out gate with a dual-rail logic circuit are the same principle, so they can transform the DNA signal strand into several DNA signal strands representing the same logic signal (the specific quantity can be set). The N fan-out gate’s function is to convert a domain-labeled DNA signal strand into an identical domain-labeled DNA signal strands, with a concentration N times that of the original signal strand. Through the fan-out gate with domain label conversion, the output strands can react with different encapsulated logic modules which are relatively independently packaged. This ensures that the logic inside the DNA system is encapsulated, as are the combined DNA systems, and the entire DNA system is at a steady state. The 2-fan out gate with a domain label is shown in Figure 7.

**Figure 8:**
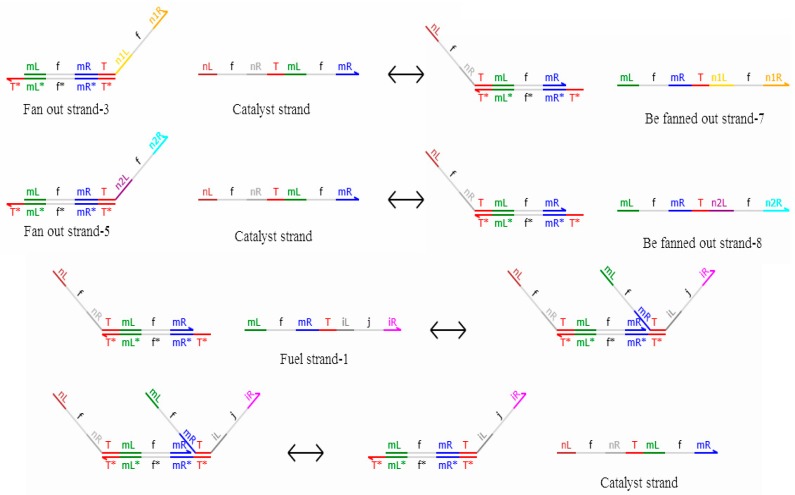
Reactions among fan out strand-3, fan out strand-5, and fuel strand-1.

It can be seen from Figure 7 that the 2-fan out gate with a domain label is composed of the domain-labeled DNA double-stranded DNA and the domain-labeled DNA single-stranded DNA, that is, fan out strand-1, fan out strand-2, fan out strand-3, fan out strand-4, fan out strand-5, fan out strand-6, fuel strand-1, and fuel strand-2. The reactions are shown in Figure 8.

In the reaction shown in Figure 8, the signal strand with domain label <nL^ f nR^ T^ mL^ f mR^> is equivalent to the catalyst. The reactions of fan out strand-3, fan out strand-5, and fuel strand-1 represent be the final outputs of fan out strand-7 and fan out strand-8, which have a logic value of 0. Similarly, when fan out strand-4, fan out strand-6, and fuel strand-2 participate in the reaction, strands <mL^ t mR^ T^ n1L^ t n1R^> and <mL^ t mR^ T^ n2L^ t n2R^> are the final outputs, and the logic value of the output strands is 1. The concentration of the output signal stand is determined by the concentration of the domain-labeled DNA double strands.

### 3.4. Reporter Gate with Domain Label

**Figure 9:**
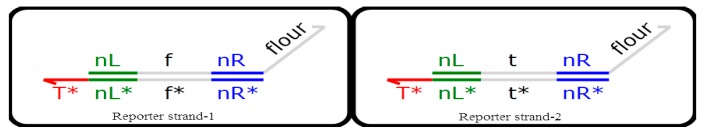
Reporter gate with a domain label.

**Figure 10:**
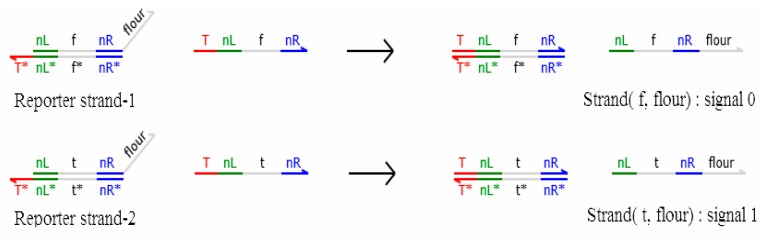
Domain label signal detection reaction.

In this paper, the experimental results are tested via the reporter gate with a domain label. The detection gate is composed of fluorophore and quenchers, which converts the DNA signal strands into fluorescent signal strands. Fluorescent signal strands are released when the reporter gate with a domain label reacts with DNA signal strands, and the fluorescent signal strands do not react with other logic gates. As shown in Figure 9, the reaction between the reporter gate with a domain label and the DNA signal strand with a domain label is shown in Figure 10.

As can be seen in Figure 9, the reporter gate with a domain label consists of reporter strand-1 and reporter strand-2, which respectively convert the corresponding domain-labeled DNA signal strands into fluorophore signal strands for detection.

In Figure 10, the logic value of the DNA signal strand can be judged by detecting strand (f, flour) or (t, flour), namely, the logic value of strand (t, flour) is 1, and the logic value of strand (f, flour) is 0.

## 4. Simulation

### 4.1. One-Bit Full Adder with Domain Label

**Figure 11:**
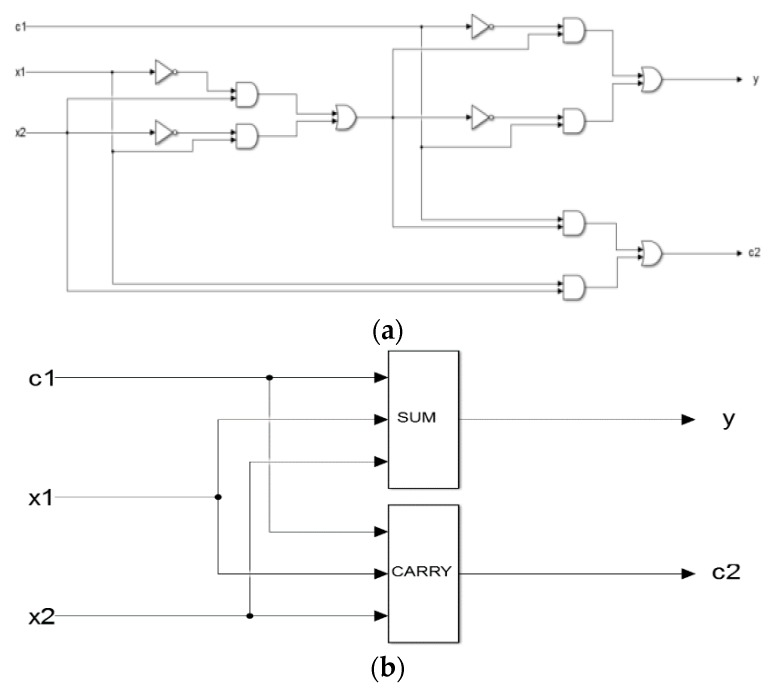
(**a**). Single-rail one-bit full adder; (**b**). One-bit full adder with a domain label.

The one-bit full adder takes the adjacent low-order carry into account. The one-bit full adder has DNA input strands denoted by c1 (low-order carry), x1, and x2, respectively, and has two DNA output strands denoted by y (sum) and c2 (carry). It can implement the addition of three binary logic values and simulate an electronic one-bit full adder. The logic circuit is shown in Figure 11a. The logic circuit of the one-bit full adder with a domain label is shown in Figure 11b.

The single-rail one-bit full adder consisting of six AND gates, three OR gates, and four NOT gates, as shown in Figure 11a, has six layers. It operates on three inputs and produces two outputs.

### 4.2. Simulation of the 1-Bit Full Adder with a Domain Label.

The 1-bit binary adder constructed by two 3-input mapping modules, which consists of 1 XOR gates with domain labels, 3 amplification gates with domain labels, one 2-fan out gate with a domain label, and reporter gates with domain labels. It can implement an addition between the 1-bit binary $A_{0}$ and 1-bit binary $B_{0}$, and finally, outputs a 2-bit binary number, $S_{1}S_{0}\;$($S_{1}$ is the output carry-bit).

**Figure 12:**
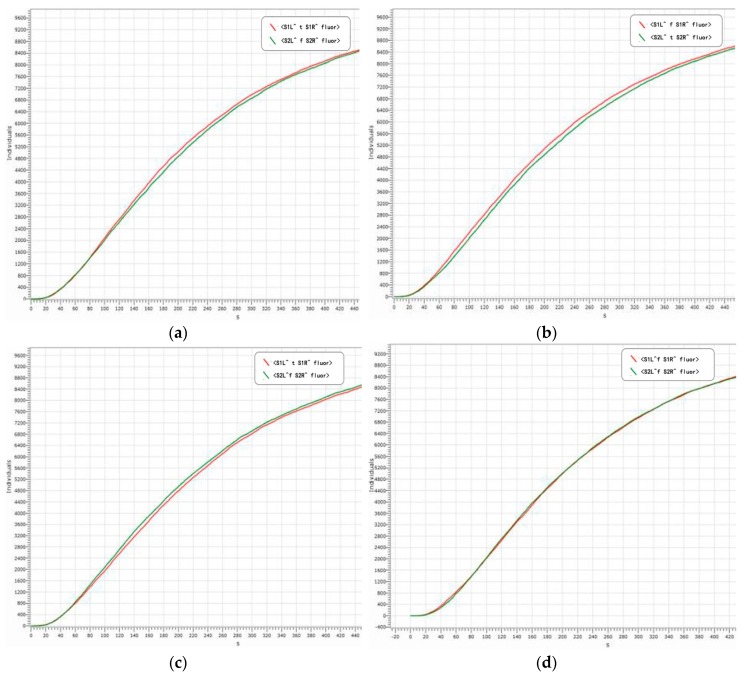
(**a**): $A_{0}=1,B_{0}=0,S_{0}=1,S_{1}=0$; (**b**): $A_{0}=1,B_{0}=1,S_{0}=0,S_{1}=1$; (**c**): $A_{0}=0,B_{0}=1,S_{0}=1,S_{1}=0$; (**d**): $A_{0}=0,B_{0}=0,S_{0}=0,S_{1}=0$.

The reporter gates with domain labels are <S1L^ _ S1R^ fluor> and <S2L^ _ S2R^ fluor>, which correspond to the summation-bit and carry-bit respectively. Specifically, when the 1-bit binary numbers are $A_{0}=1$ and $B_{0}=0$, the corresponding results are $S_{0}=1$ and $S_{1}=0$. Specifically, the output strands are <S1L^ t S1R^ fluor> and <S2L^ f S2R^ fluor>. When the 1-bit binary numbers are $A_{0}=1$ and $B_{0}=1$, the corresponding results are $S_{0}=0$ and $S_{1}=1$. Specifically, the output strands are <S1L^ f S1R^ fluor> and <S2L^ t S2R^ fluor>. When the 1-bit binary numbers are $A_{0}=0$ and $B_{0}=1$, the corresponding results are $S_{0}=1$ and $S_{1}=0$. Specifically, the output strands are <S1L^ t S1R^ fluor> and <S2L^ f S2R^ fluor>. When the 1-bit binary numbers are $A_{0}=0$ and $B_{0}=0$, the corresponding results are $S_{0}=0$ and $S_{1}=0$. Specifically, the output strands are <S1L^ f S1R^ fluor> and <S2L^ f S2R^ fluor>. The simulation results are shown in Figure 12.

From Figure 12, the following conclusions can be drawn. Firstly, the logical values of the reaction results in Figure 12a–d are correct, in accordance with the logical operation of binary summation, indicating that the adder with a domain label is feasible and it has a high accuracy. Secondly, the entire reaction curve is smooth and the reaction process is very stable, which indicating the stability of the adder with a domain label is improved, so the reaction state can be determined. Thirdly, the reaction reaches a state of equilibrium in about 540 s and the sensitivity is higher. In summary, the accuracy, stability, and sensitivity responses of the adder with a domain label satisfy our experimental requirements, thus providing a new perspective for the construction of other bio-circuits.

**Figure 13:**
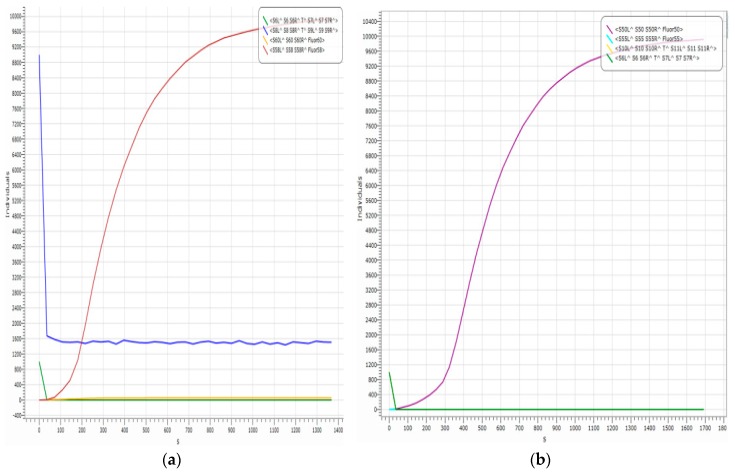
(**a**): $A_{0}^{1}=1,B_{0}^{0}=1,S_{0}^{1}=1,S_{1}^{0}=1$; (**b**): $A_{0}^{1}=1,B_{0}^{1}=1,S_{0}^{1}=0,S_{1}^{1}=1$.

To further verify the advantages of the adder with a domain label, we simulate the double-rail 1-bit adder when the threshold concentration in the double-rail logic is at the extreme edge, as shown in Figure 13.

In Figure 13, the reporter gates are <S60L S60 S60R Fluor01> (SM^1), <S50L S50 S50R Fluor00> (SM^0), <S55L S55 S55R Fluor11> (CY^1), and <S58L S58 S58R Fluor10> (CY^0), which correspond, respectively, to $S_{0}^{1}=1,{\;S}_{0}^{0}=1,{\;S}_{1}^{1}=1,S_{1}^{0}=1$. The 1-bit binary numbers A and $B_{0}$ are converted into $A_{0}^{0},{\;A}_{0}^{1},{\;B}_{0}^{0},B_{0}^{1}$. Their DNA input strands correspond to <S4L^ S4 S4R^ T^ S5L^ S5 S5R^>, <S6L^ S6 S6R^ T^ S7L^ S7 S7R^>, <S8L^ S8 S8R^ T^ S9L^ S9 S9R^>, and <S10L^ S10 S10R^ T^ S11L^ S11 S11R^>.

In theory, when the 1-bit binary numbers are $A_{0}^{1}=1$ and $B_{0}^{0}=1$, the corresponding results are $S_{0}^{1}=1$ and $S_{1}^{0}=0$; specifically, when the input strands are <S6L^ S6 S6R^ T^ S7L^ S7 S7R^> ($(A_{0}^{1}$) $A_{0}^{1}$ and <S8L^ S8 S8R^ T^ S9L^ S9 S9R^> ($B_{0}^{0}$), the output strands are <S60L S60 S60R Fluor01> (SM^1) and <S58L S58 S58R Fluor10> (CY^0). In other words, the logic values of them are both 1. When the 1-bit binary numbers are $A_{0}^{1}=1$ and $B_{0}^{0}=1$ the corresponding results are $S_{0}^{0}=1$ and $S_{1}^{1}=1.$ Specifically, when the input strands are <S6L^ S6 S6R^ T^ S7L^ S7 S7R^> ($A_{0}^{1}$) and <S10L^ S10 S10R^ T^ S11L^ S11 S11R^> ($B_{0}^{1}$), the output strands are <S50L S50 S50R Fluor00> (SM^0) and <S55L S55 S55R Fluor11> (CY^1), which both have logic values of 1.

However, the actual logic operations in Figure 13a,b are wrong. Specifically, in Figure 13a, when the input strands are <S6L^ S6 S6R^ T^ S7L^ S7 S7R^> ($A_{0}^{1}$) and <S8L^ S8 S8R^ T^ S9L^ S9 S9R^> ($B_{0}^{0}$), the logic value of output strand <S58L S58 S58R Fluor10> (CY^0) is 1, while the logic value of output strand <S60L S60 S60R Fluor01> (SM^1) is 0, which goes against the binary logic algorithms. In Figure 13b, when the input strands are <S6L^ S6 S6R^ T^ S7L^ S7 S7R^> ($A_{0}^{1}$) and <S10L^ S10 S10R^ T^ S11L^ S11 S11R^> (${\;B}_{0}^{1}$), the logic value of output strand <S50L S50 S50R Fluor00> (SM^0) is 1, while the logic value of output strand<S55L S55 S55R Fluor11> (CY^1) is 0, which goes against the binary logic algorithms.

The reason for the logic error in the reactions shown in Figure 13 is the threshold concentration in the double-rail logic Seesaw gate. In general, a DNA signal strand with a unit concentration of 0.9 to 1 represents logic value 1, and a DNA signal strand with a 0 to 0.1 unit density represents a logic value of 0. However, the threshold cannot strictly distinguish between high and low concentrations when its concentration is at 0.1~0.2 units or 0.8~0.9 units, resulting in the wrong signal being output (Figure 13). And the logic circuit with domain t and domain f is determined throughout the entire reaction process (Figure 11), which provides the potential for molecular automation, such as DNA 4 × 4 multiplier operations, n-bit addition, and so on.

### 4.3. Simulation of DNA 4 × 4 Multiplier with Domain Label

**Figure 14:**
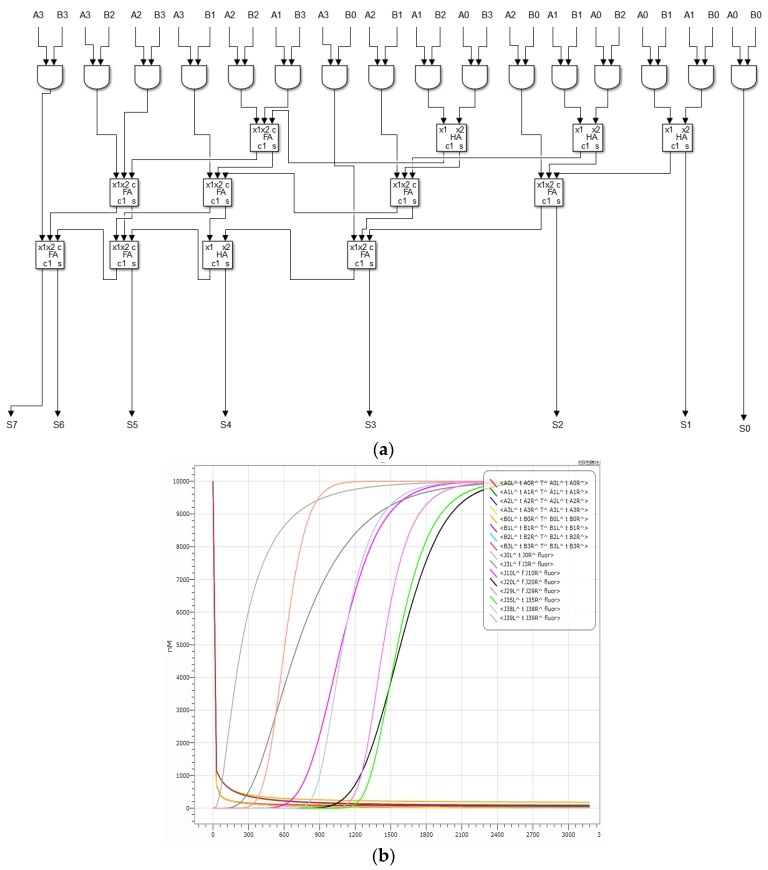
(**a**): Binary DNA 4 × 4 multiplier with a domain label; (**b**): Simulation of DNA 4 × 4 multiplier with a domain label.

Figure 14a shows a domain-labeled binary DNA 4 × 4 multiplier based on DNA strand permutation, which consists of 16 domain-labeled AND gates, four one-half adders (without detection gates), and eight one-bit full adders (without detection gates). Eight domain-labeled detection gates are composed. Of course, domain-labeled amplifiers can also be added at other desired locations. Since the domain-labeled fluorescent signal chain cannot react with other logic gates just to facilitate detection, the removal of the detection gate does not affect the result of the reaction, so detection gates are removed from semi-adder and full adder, and only the A domain-labeled detection gate is added to the final output of the multiplier to facilitate the detection of the eight domain tag output signal strands.

Figure 14b is a simulation of a binary DNA 4 × 4 multiplier, simulated with 1111 × 1111 = 11100001 as an example. Table 2 shows the logical values of the DNA input and output strands.

The concentration of the eight domain-labeled DNA signal strands rapidly decreases to less than 0.1 times the unit concentration within the initial 60 s, and then slowly decreases to almost zero over the remaining time period; the concentration of the domain-labeled fluorescent signal chain rapidly rose to 0.9 times the unit concentration or more within 2100 s, and then it slowly rose until it was very close to a concentration of 1 unit. The domain-labeled binary DNA 4 × 4 multiplier can realize the multiplication of 4-bit binary number and 4-bit binary number. The whole reaction is very stable, which indicates that the designed domain-labeled binary DNA 4 × 4 multiplier has good stability and encapsulation, which further demonstrates that the AND gate, OR gate, NOT gate, amplifier, fan-out gate, and detection gate with the domain label have good stability and encapsulation, which lays the foundation for the realization of DNA computers.

### 4.4. Simulation of 8-Bit Binary Adder/Subtractor with Domain Label

Similar to the 1-bit full adder, the 8-bit adder takes the adjacent low-order carry into account. It is constructed by 1-mapping modules, 2-mapping modules, and 3-mapping modules, and consists of eight XOR gates with a domain label, eight one-bit full adders with a domain label, 24 amplification gates with a domain label and one 9-fan out gate with a domain label. It can implement an adder or subtractor between the 8-bit binary $A_{7}A_{6}A_{5}A_{4}A_{3}A_{2}A_{1}A_{0}$ and the 8-bit binary $B_{7}B_{6}B_{5}B_{4}B_{3}B_{2}B_{1}B_{0}$, and finally, outputs a 9-bit binary number $S_{8}S_{7}S_{6}S_{5}S_{4}S_{3}S_{2}S_{1}S_{0}$ ($S_{8}$ is the output carry-bit). It has 16 input DNA strands with domain labels, nine DNA output strands with domain labels, and one DNA switch strand with a domain label (denoted by A#S) which decides whether to implement an 8-bit adder or 8-bit subtractor (when A#S = 0, the DNA 8-bit adder is used, when A#S = 1, the DNA 8-bit subtractor is used). The logical values corresponding to the DNA input and output strands with domain labels are shown in Table 1 (take 00101101−10010110= 010010111 as an example).

The single logic circuit discussed below has eight inputs and eight outputs. $A_{i},B_{i}$ and $C_{i}$ represent the ith input and the ith output respectively. The logic function expressions of the single-rail logic circuit are as follows:

$$\begin{array}{l} S_{i}=A_{i}(A_{0},A_{1},\ldots ,A_{7})+B_{i}(B_{0},B_{1},\ldots ,B_{7})\\ =f_{i}(A_{0},A_{1},\ldots ,A_{7},{\bar{A}}_{0},{\bar{A}}_{1},\ldots ,{\bar{A}}_{7})+g_{i}(B_{0},B_{1},\ldots ,B_{7},{\bar{B}}_{0},{\bar{B}}_{1},\ldots ,{\bar{B}}_{7}) \end{array}$$

**Figure 15:**
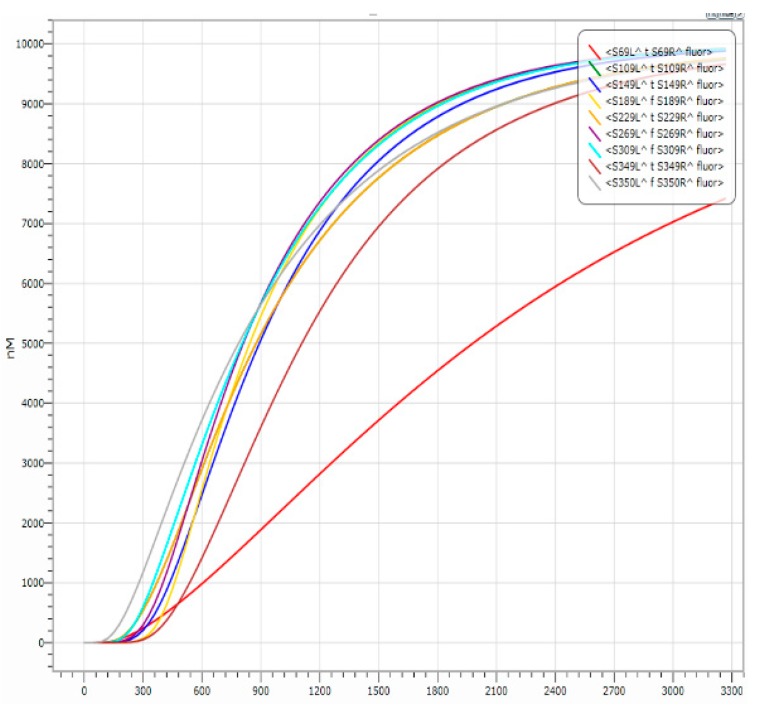
Simulation of 00101101 − 10010110 = 010010111.

In Table 3, the decimal number 77 minus the decimal number 150 is equal to the negative decimal number 73. Based on the binary complement operation, the logic expression is $77+(2^{8}-150)=77+106=183$, and the decimal number 183 corresponding to the binary number is 10110111. Obviously, the complement of the negative decimal number 73 is the binary number 10110111. So, the 8-bit binary adder/subtractor correctly calculates the result of subtracting two 8-bit binary numbers, namely, 00101101−10010110= 010010111. The simulation of it is shown in Figure 15.

In Figure 15, the function of the 8-bit binary subtractor with a domain label is realized; in the input 8-bit binary code, the meaning of 00101101 is 77, and the meaning of 10010110 is 150. That is, the reaction input 00101101 − 10010110 will output 010010111. If we input strands with the domain label <S2L^ t S2R^ T^ S2L^ t S2R^> … <S9L^ f S9R^ T^ S9L^ f S9R^> and strands with the domain label <S10L^ f S10R^ T^ S10L^ f S10R^> … <S17L^ t S17R^ T^ S17L^ t S17R^> at the same time, we do not forget the strands <S0L^ t S0R^ T^ S0L^ f S0R^> and <S0L^ t S0R^ T^ S0L^ t S0R^>, which determine the addition and subtraction of the reaction. When the reaction input <S0L^ t S0R^ T^ S0L^ t S0R^>, the reaction is a subtraction, and it outputs strands with domain labels <S69L^ t S69R^ fluor>, <S109L^ t S109R^ fluor>, <S149L^ t S149R^ fluor>, <S189L^ f S189R^ fluor>, <S229L^ t S229R^ fluor>, <S269L^ f S269R^ fluor>, <S309L^ f S309R^ fluor>, <S349L^ t S349R^ fluor>, and <S350L^ f S350R^ fluor>. Otherwise, the reaction performs an addition operation. The entire reaction correctly calculates the subtraction of two 8-bit binary numbers, and the entire reaction occurs quickly and orderly.

### 4.5. Summary

The logic circuit constructed by a 1-mapping module, 2-mapping module and 3-mapping module has a lower design complexity, a shorter calculation time, and higher stability than the dual-rail logic circuit. Regarding the complexity of the design, firstly, the 1-mapping module and the 2-mapping module are combined to simulate the correctness of the one-bit full adder with the domain label, and then, the n-bit stable logic circuit is constructed. The fewer layers used, the higher the parallelism is, and the shorter the computing time is. Regarding the stability, logic values of 1 and 0 in the dual-rail logic circuit are represented by a higher concentration and a lower concentration, respectively; however, thresholding strands may not distinguish DNA signal strands with higher concentrations from DNA signal strands with lower concentrations, which introduces an error signal into the DNA reaction. Logic values of 1 and 0 in the logic circuit constructed by mapping modules are represented by the domain labels t and f. This is deterministic and does not make DNA reaction produce an error signal. In addition, the standard deviation of the computation time of the logic circuit constructed by mapping modules is far less than that of the dual-rail logic circuit, indicating that mapping modules possess stability and logic circuits constructed by them are more stable.

## 5. Conclusions

Basic logic gates, an amplification gate, a fan-out gate, a reporter gate with a domain label (domains t and f), and N-mapping modules with domain labels were designed in this paper. The mapping modules included a 1-mapping module, a 2-mapping module, a 3-mapping module and an N-mapping module according to how many inputs they operated on. DNA logic circuits constructed with a 1-mapping module, a 2-mapping module, a 3-mapping module and an N-mapping module were shown to possess a lower design complexity, fewer layers, higher parallelism, higher stability, and shorter time complexity, which was verified through a comparison with a one-bit full adder with a domain label. A DNA 8-bit adder/subtractor was designed with mapping modules; this could be applied to design more stable and faster DNA computers in the future, so that more and more NP-complete problems can be solved with shorter time complexity.

## Figures and Tables

**Table 1:** Double strands of the 2-input mapping module.

Identification	DNA Strands	Simple Note
n1∈{t,f}	{T^*}[mL^ f mR^ T^]:[nL^ f nR^ T^]<hL^ n1 hR^>	(ff, n1)
n2∈{t,f}	{T^*}[mL^ f mR^ T^]:[nL^ t nR^ T^]<hL^ n2 hR^>	(ft, n2)
n3∈{t,f}	{T^*}[mL^ t mR^ T^]:[nL^ f nR^ T^]<hL^ n3 hR^>	(tf, n3)
n4∈{t,f}	{T^*}[mL^ t mR^ T^]:[nL^ t nR^ T^]<hL^ n4 hR^>	(tt, n4)

**Table 2:** Logic values of DNA signal strands (1111 × 1111 = 11100001).

DNA Strands with Domain Labels	Input/Output	Logic Value
<A0L^ t A0R^ T^ A0L^ t A0R^>	A0	1
<A1L^ t A1R^ T^ A1L^ t A1R^>	A1	1
<A2L^ t A2R^ T^ A2L^ t A2R^>	A2	1
<A3L^ t A3R^ T^ A3L^ t A3R^>	A3	1
<B0L^ t B0R^ T^ B0L^ t B0R^>	B0	1
<B1L^ t B1R^ T^ B1L^ t B1R^>	B1	1
<B2L^ t B2R^ T^ B2L^ t B2R^>	B2	1
<B3L^ t B3R^ T^ B3L^ t B3R^>	B3	1
<J0L^ t J0R^ fluor>	S0	1
<J3L^ f J3R^ fluor>	S1	0
<J10L^ f J10R^ fluor>	S2	0
<J20L^ f J20R^ fluor>	S3	0
<J29L^ f J29R^ fluor>	S4	0
<J35L^ t J35R^ fluor>	S5	1
<J38L^ t J38R^ fluor>	S6	1
<J39L^ t J39R^ fluor>	S7	1

**Table 3:** Logic values of DNA signal strands (00101101 − 10010110 = 010010111).

DNA Strands with Domain Label	Input/Output	Logic Value
<S2L^ t S2R^ T^ S2L^ t S2R^>	$A_{0}$	1
<S3L^ f S3R^ T^ S3L^ f S3R^>	$A_{1}$	0
<S4L^ t S4R^ T^ S4L^ t S4R^>	$A_{2}$	1
<S5L^ t S5R^ T^ S5L^ t S5R^>	$A_{3}$	1
<S6L^ f S6R^ T^ S6L^ f S6R^>	$A_{4}$	0
<S7L^ t S7R^ T^ S7L^ t S7R^>	$A_{5}$	1
<S8L^ f S8R^ T^ S8L^ f S8R^>	$A_{6}$	0
<S9L^ f S9R^ T^ S9L^ f S9R^>	$A_{7}$	0
<S10L^ f S10R^ T^ S10L^ f S10R^>	$B_{0}$	0
<S11L^ t S11R^ T^ S11L^ t S11R^>	$B_{1}$	1
<S12L^ t S12R^ T^ S12L^ t S12R^>	$B_{2}$	1
<S13L^ f S13R^ T^ S13L^ f S13R^>	$B_{3}$	0
<S14L^ t S14R^ T^ S14L^ t S14R^>	$B_{4}$	1
<S15L^ f S15R^ T^ S15L^ f S15R^>	$B_{5}$	0
<S16L^ f S16R^ T^ S16L^ f S16R^>	$B_{6}$	0
<S17L^ t S17R^ T^ S17L^ t S17R^>	$B_{7}$	1
<S0L^ t S0R^ T^ S0L^ t S0R^>	A#S	1
<S69L^ t S69R^ fluor>	$S_{0}$	1
<S109L^ t S109R^ fluor>	$S_{1}$	1
<S149L^ t S149R^ fluor>	$S_{2}$	1
<S189L^ f S189R^ fluor>	$S_{3}$	0
<S229L^ t S229R^ fluor>	$S_{4}$	1
<S269L^ f S269R^ fluor>	$S_{5}$	0
<S309L^ f S309R^ fluor>	$S_{6}$	0
<S349L^ t S349R^ fluor>	$S_{7}$	1
<S350L^ f S350R^ fluor>	$S_{8}$	0
